# Preprocedural prediction of non-curative endoscopic submucosal dissection for early gastric cancer

**DOI:** 10.1371/journal.pone.0206179

**Published:** 2018-10-24

**Authors:** Hyeong Seok Nam, Cheol Woong Choi, Su Jin Kim, Dae Hwan Kang, Hyung Wook Kim, Su Bum Park, Dae Gon Ryu, Jung Sik Choi

**Affiliations:** 1 Department of Internal Medicine, Pusan National University School of Medicine and Research Institute for Convergence of Biomedical Science and Technology, Pusan National University Yangsan Hospital, Yangsan, Korea; 2 Department of Internal Medicine, Inje University College of Medicine, Busan Paik Hospital, Pusan, Korea; Universitá Sapienza di Roma, ITALY

## Abstract

**Background and aim:**

Endoscopic submucosal dissection (ESD) has been accepted as the treatment of choice for early gastric cancer (EGC) without lymph node metastasis. However, additional surgical gastrectomy should be considered after non-curative endoscopic resection. We aimed to evaluate the predictive factors associated with non-curative endoscopic resection.

**Methods:**

Between November 2008 and June 2015, a retrospective study was conducted in a single, tertiary, referral hospital. A total of 596 EGC lesions resected by ESD were analyzed. Non-curative endoscopic resection was defined as the occurrence of lesions associated with piecemeal resection, positive resection margins, lymphovascular invasion, or lesions that did not meet the expanded indications for ESD.

**Results:**

The rate of non-curative endoscopic resection was 16.1%. The mean follow-up period was 35.3 ± 25.0 months. Associated predictive factors for non-curative endoscopic resection were female sex (OR, 2.470; *p* = 0.004), lesion size ≥ 20 mm (OR 3.714; *p* < 0.001), longer procedure time (OR 2.449, p = 0.002), ulceration (OR 3.538, p = 0.002), nodularity (OR 2.967, p<0.001), depression (OR 1.806, p = 0.038), undifferentiated carcinoma (OR 2.825, p = 0.031) and lesion located in the mid or upper third of stomach (OR 7.135 and OR 4.155, p<0.001, respectively). As the number of risk factors increased, the risk of non-curative ESD also increased.

**Conclusions:**

Prior to selection of ESD, the risks associated with non-curative ESD should be considered so that appropriate treatment modalities may be selected.

## Introduction

Gastric cancer that is confined to the mucosa or submucosa is defined as early gastric cancer (EGC) regardless of lymph node metastasis [1]. With the development of endoscopic instruments and techniques, endoscopic submucosal dissection (ESD) has been accepted as the treatment of choice for EGC without lymph node metastasis. Before introduction of ESD, conventional endoscopic mucosal resection (EMR) techniques using snare comprised the standard approach, but they have technical limitations for EGCs larger than 20 mm in diameter or submucosal fibrosis. After introduction of ESD, the en-bloc resection rate of EGC regardless of lesion size is higher than that observed with EMR [2]. The reported 5-year survival of ESD for EGC, which met the absolute and expanded indications, was over 95% [3,4]. According to the National Cancer Screening program of the republic of Korea, Korean adults over 40 years can receive endoscopic gastric cancer screening every other year. By 2012, gastric cancer screening rates reached 70% in the Republic of Korea [5].

The important advantage of ESD compared with surgical gastrectomy may be a better quality of life by avoiding gastrectomy. Curative endoscopic resection can be assessed based on the meticulous pathologic examination of the resected specimen. The Japanese Gastric Cancer Association defined a curative resection as an en-bloc resection of EGC that showed no marginal involvement and met the expanded indication [6]. Despite the higher en-bloc resection rate, up to 16.5% of patients who underwent ESD were reported to have a non-curative endoscopic resection [7]. After non-curative endoscopic resection, additional surgical treatment should be performed because of the high risk of local recurrence or lymph node metastasis.

To avoid unnecessary endoscopic treatment, it may be important to predict the occurrence of non-curative endoscopic resection before treatment selection. In the present study, we aimed to evaluate the predictive factors associated with non-curative pathologic results after ESD and the clinical outcomes of non-curative endoscopic resection patients during follow-up examinations.

## Methods

### Patients

**Fig 1:**
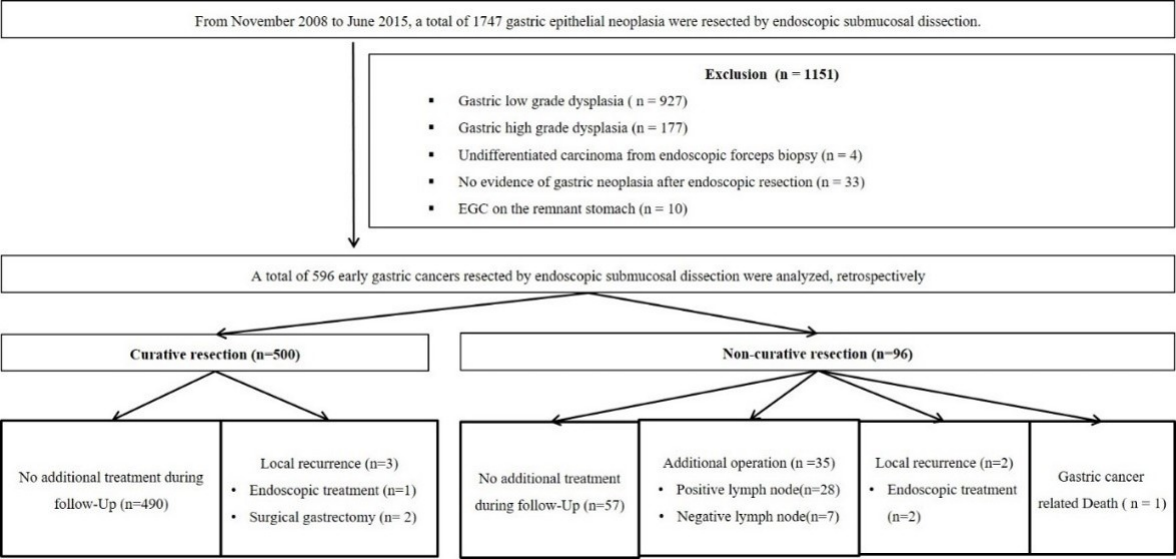
Study flow. ESD, endoscopic submucosal dissection; EGC, early gastric cancer.

From November 2008 to June 2015, the medical records of patients who underwent ESD were retrospectively reviewed at the Pusan National University Yangsan Hospital in the Republic of Korea. During the study period, a total of 1747 gastric epithelial neoplasias were resected by ESD. In our institution, the patients who met either the absolute or expanded indications before ESD were recommended for ESD. The following lesions were excluded from the present study; EGC on the remnant stomach (n = 10), undifferentiated carcinoma from endoscopic forceps biopsy (n = 4), gastric low grade dysplasia (n = 927), gastric high grade dysplasia (n = 177) and no evidence of gastric neoplasia after endoscopic resection (n = 33). After exclusion, a total of 596 EGCs, resected by ESD, were enrolled and analyzed (Fig 1). All procedures followed were in accordance with the ethical standards of the responsible committee on human experimentation and with the Helsinki Declaration of 1964 and later versions. Informed consent was impossible because of the retrospective nature. Only data from the patients were analyzed retrospectively, and information that would identify of patients was not included. The study was approved by the Ethics Committee of our Institutional Review Board (05-2018-035).

### Procedure

During the ESD procedure, conscious sedation using intravenous midazolam (0.05 mg/kg) and pethidine (50 mg) was performed. We used two types of electrosurgical knifes: a needle or an insulation-tipped electrosurgical knife. All patients were placed in the left, lateral, decubitus position and examined with either a standard single-channel endoscope (GIF-H260, GIF-H260Z or GIF-HQ290; Olympus Optical, Tokyo, Japan) or a 2-channel endoscope (GIF-2TQ260M; Olympus Optical, Tokyo, Japan). Before the ESD procedure, the lateral margin of the lesion was determined using either indigo carmine spray or an image-enhanced endoscope with narrow band imaging. After determination of the margin of the EGC, a marking with the electrosurgical knives was created 1–2 mm outside the lesion. After marking, a solution containing a mixture of normal saline, epinephrine, and indigo carmine was injected into the submucosa to raise the lesion from muscularis propria. After submucosal injection, a circumferential incision and submucosal dissection was performed using electrosurgical knives. After complete resection, preventive coagulation was implemented for all visibly exposed vessels in the artificial ulcer bed.

After successful ESD, we recommend regular follow-up examinations 2–3 months after the initial ESD and every 6 months thereafter. After 2 years of follow-up, for patients without evidence of either recurrence or metachronous lesions, an annual endoscopic examination is recommended. For patients with non-curative endoscopic resection results, we recommended surgical gastrectomy. However, if the non-curative resection was associated with a piecemeal resection of a differentiated carcinoma that was confined to the mucosa without evidence of lymphovascular invasion, or an en-bloc resected differentiated carcinoma with lateral margin involvement of the short segment, non-surgical treatment options, such as repeated endoscopic resection or endoscopic destructive therapy using an argon plasma coagulation, were considered after obtaining the patient’s informed consent.

### Definitions

**Fig 2:**
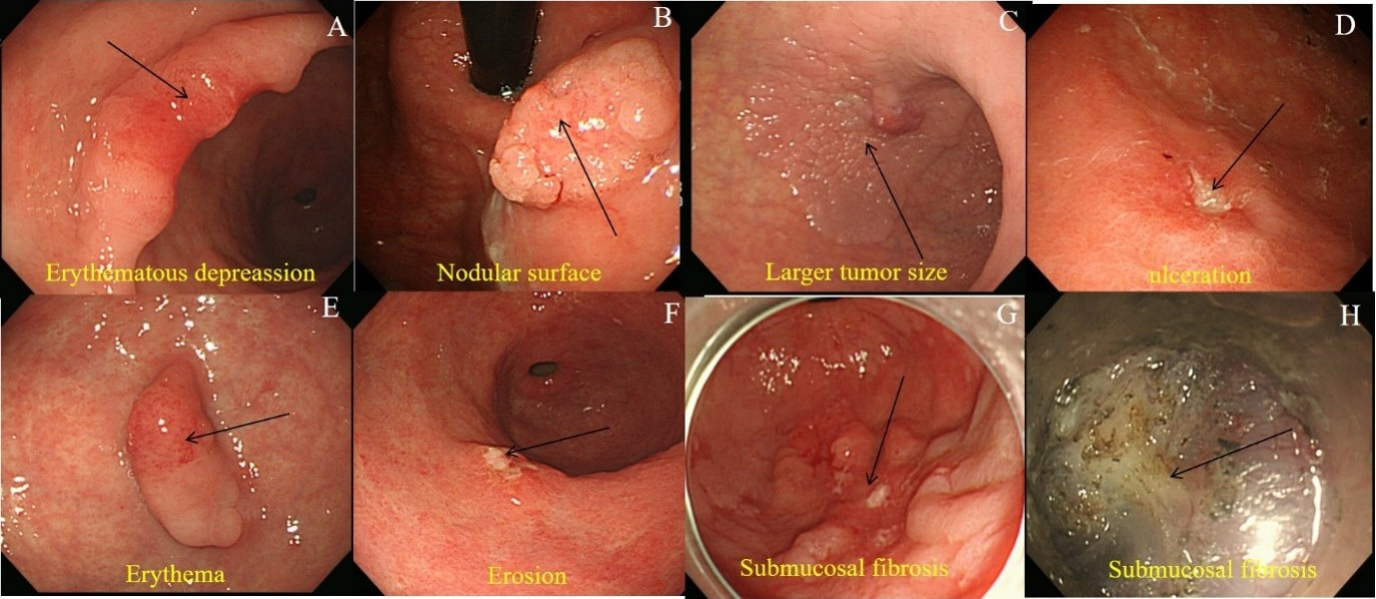
Endoscopic characteristics. (A) surface depression. (B) Nodular surface. (C) larger tumor size (≥ 20mm), (D) Ulceration. (E) Erythematous color change. (F) Erosion. (G) Central ulceration with depressed surface. (H) During endoscopic dissection, submucosal fibrosis was noted.

All endoscopic photographs and medical records were reviewed by one endoscopist (CW Choi M.D., Ph.D.). The endoscopic macroscopic appearances of the lesions were classified according to the Paris Classification (elevated, flat, and depressed) [8]. The location of the EGC was classified as the lower third, mid, or upper third of the stomach according to the Japanese Classification of Gastric Cancer [9]. The size of the EGC was measured by pathological examination (maximal diameter). Erythema and discoloration were determined after comparing the color of the EGC with the background normal mucosa. Nodularity was measured as the presence of irregularly raised or nodular mucosa. The submucosal fibrosis was recorded after confirming the presence of fibrosis while dissecting the submucosa (Fig 2). We measure the endoscopic extent of atrophic gastritis using the Kimura and Takemoto classification system: mild (normal to closed type 2), moderate (closed type 3 to open type 1), and severe (open type 2 to open type 3) [10]. The procedure time was calculated from the marking to the completion of preventing coagulation after the removal of the EGC.

The EGC was histologically classified as differentiated or undifferentiated carcinoma according to the Japanese Classification of Gastric Carcinoma [9]. The resected specimens were stretched, pinned, and fixed with formalin. Specimens that were resected in a piecemeal fashion were reconstructed as accurately as possible. Fixed specimens were then sectioned at 2 mm intervals. Endoscopic resection of a lesion as a single piece was defined as an en-bloc resection. Endoscopic complete resection was defined as the absence of tumors cells at the margins of an en-bloc resected specimen. The endoscopic resection was determined to be curative when the EGCs met the expanded indication and the lesion was completely endoscopically resected, without evidence of lymphovascular invasion [6]. Resections that did not satisfy the criteria for curative endoscopic resection were considered to be non-curative endoscopic resections.

### Statistical analysis

Univariate analysis using either a chi-square test or the Fisher’s exact test for categorical variables, or the Student’s t-test for continuous variables was performed. The variables with p < 0.05 in the univariate analysis were included for the multivariable analysis using multiple logistic regression models. *P* < 0.05 was considered to be statistically significant. Calculations were performed using the Statistical Package for the Social Sciences (SPSS) version 21.0 for Windows (IBM Corp., Armonk, NY, USA).

## Results

### Baseline characteristics of patients who underwent ESD

A total of 596 patients with EGCs who treated by ESD were analyzed. The en-bloc resection and complete resection rates were 98.3% and 93.8%, respectively. The final non-curative resection rate was 16.1% (96/596). The patient population was predominantly male (78.4%), with a mean age of 69.5 ± 9.6 years. The mean tumor size was 14.1 ± 8.8 mm. The mean procedure time was 27.1 ± 18.3 min. The mean follow-up length was 35.3 ± 25.0 months. The predominant location of the primary lesions was in the lower third of the stomach (69.0%). The most common gross type was the depressed type (59.1%) (Table 1). Moderate extent of atrophic gastritis was common (47.3%). The most common histology of EGC was the differentiated EGC (97.6%) (Table 1).

**Table 1:** Baseline characteristics and comparative analysis of lesions between curative and non-curative endoscopic resection.

	Non-curative endoscopic resection (n = 96)	Curative endoscopic resection (n = 500)	Total (n = 596)	P value
**Age, years, mean (SD)**	71.0 (10.7)	69.2 (9.3)	69.5 (9.6)	0.102
**Sex, male, n (%)**	67 (69.8)	400 (80.0)	467 (78.4)	0.026
**Lesion size, mm, mean (SD)**	22.2 (12.8)	12.6 (6.8)	14.1 (8.8)	<0.001
**Lesion size ≥ 20 mm, n (%)**	49 (51.0)	73 (14.6)	122 (20.5)	<0.001
**Procedure time, min, mean (SD)**	40.7 (25.3)	24.5 (15.3)	27.1 (18.3)	<0.001
**Procedure time ≥ 30 min, n (%)**	68 (70.8)	149 (29.8)	217 (36.4)	<0.001
**Follow up, month, mean (SD)**	34.2 (23.9)	35.5 (25.2)	35.3 (25.0)	0.643
**Locations of lesions, n (%)**				<0.001
Lower third	46 (47.9)	365 (73.0)	411 (69.0)	
Mid third	29 (30.2)	113 (22.6)	142 (23.8)	
Upper third	21 (21.9)	22 (4.4)	43 (7.2)	
**En-bloc resection, n (%)**	86 (89.6)	500 (100)	586 (98.3)	<0.001
**Complete resection, n (%)**	59 (61.5)	500 (100)	559 (93.8)	<0.001
**Pathologic diagnosis of ESD, n (%)**				<0.001
Differentiated carcinoma	85 (88.5)	487 (97.4)	572 (97.6)	
Undifferentiated carcinoma	11 (11.5)	13 (2.6)	24 (4.0)	
**Endoscopic atrophic gastritis, n (%)**				0.909
Mild	23 (24.0)	130 (26.0)	153 (25.7)	
Moderate	46 (47.9)	236 (47.2)	282 (47.3)	
Severe	27 (28.1)	134 (26.8)	161 (27.0)	
**Morphologic of lesions, n (%)**				0.365
Elevated	35 (36.5)	146 (29.2)	181 (30.4)	
Flat	9 (9.4)	54 (10.8)	63 (10.6)	
Depressed	52 (54.2)	300 (60.0)	352 (59.1)	
**Ulceration, n (%)**	20 (20.8)	41 (8.2)	61 (10.2)	<0.001
**Scar, n (%)**	19 (19.8)	69 (13.8)	88 (14.8)	0.130
**Submucosal fibrosis, n (%)**	48 (50.0)	132 (26.4)	180 (30.2)	<0.001
**Discoloration, n (%)**	6 (6.3)	16 (3.2)	22 (3.7)	0.147
**Erythema, n (%)**	92 (95.8)	479 (95.8)	571(95.8)	0.988
**Nodularity, n (%)**	48 (50.0)	130 (26.0)	178 (29.9)	<0.001
**Depression, n (%)**	57 (59.4)	213 (42.6)	270 (45.3)	0.002
**Erosion, n (%)**	29 (30.2)	229 (45.8)	258 (43.3)	0.005

SD, standard deviation; ESD, endoscopic submucosal dissection

### Predictive factors associated with non-curative endoscopic resection: Univariate and multivariate analysis

**Fig 3:**
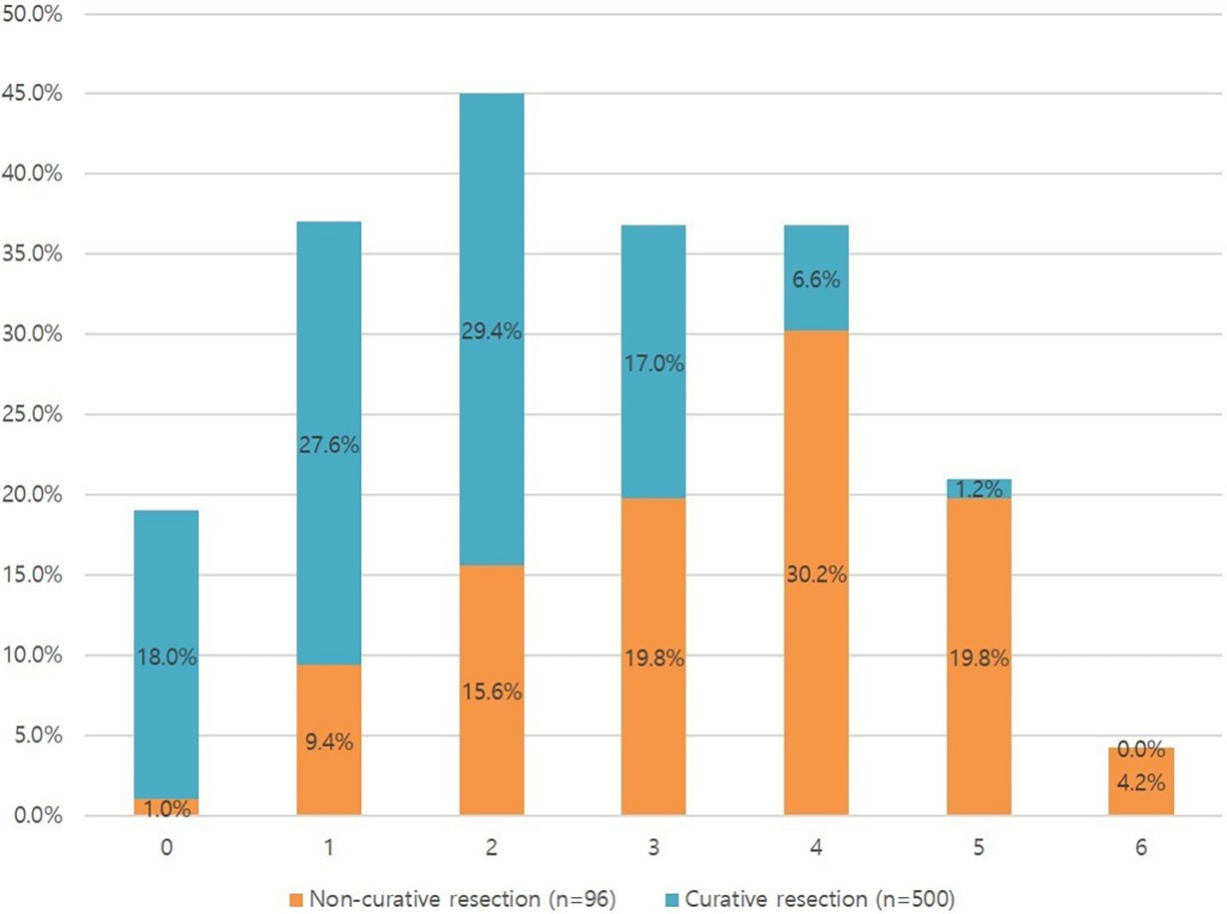
Effects of the presence of 0–7 predictive factors associated with non-curative endoscopic resection.

The factors associated with non-curative endoscopic resection were analyzed. After univariate analysis, the following variables were determined to be significant risk factors: female sex, larger lesion size (≥ 20 mm), longer procedure time (≥ 30 min), location of lesions (mid or upper third of stomach), ulceration, submucosal fibrosis, nodularity, depression, erosion, and undifferentiated carcinoma (Table 1). After multivariate analysis the following variables were determined to be significant risk factors: female sex (OR 2.470, 95% CI, 1.331–4.585, p = 0.004), larger lesion size ≥ 20 mm (OR 3.714, 95% CI, 2.103–6.556, p < 0.001), longer procedure time ≥ 30 min (OR 2.449, 95% CI, 1.393–4.304, p = 0.002), ulceration (OR 3.538, 95% CI, 1.571–7.965, p = 0.002), nodularity (OR 2.967, 95% CI, 1.689–5.211, p < 0.001), depression (OR 1.806, 95% CI, 1.034–3.153, p = 0.038), undifferentiated carcinoma (OR 2.825, 95% CI, 1.097–7.271, p = 0.031), and lesions located at the mid (OR 7.135, 95% CI, 3.106–16.388, p<0.001) or upper third (OR 4.155, 95% CI, 1.732–9.962, p<0.001) of stomach (Table 2). The number of predictive risk factors was calculated and a high number of risk factors were associated with an increased frequency of non-curative endoscopic resection (Table 3 and Fig 3).

**Table 2:** Multivariate analysis associated with non-curative endoscopic resection.

	OR	95% C.I	p value
**Female sex**	2.470	1.331–4.585	0.004
**Lesion size ≥ 20 mm**	3.714	2.103–6.556	<0.001
**Longer procedure time ≥ 30 min**	2.449	1.393–4.304	0.002
**Surface ulceration**	3.538	1.571–7.965	0.002
**Submucosal fibrosis**	1.204	0.664–2.185	0.540
**Surface nodularity**	2.967	1.689–5.211	<0.001
**Surface depression**	1.806	1.034–3.153	0.038
**Surface erosion**	1.112	0.627–1.971	0.717
**Undifferentiated carcinoma**	2.825	1.097–7.271	0.031
**Location of lesion**	1.112	0.627–1.971	-
Lower third	1.000	-	-
Mid	7.135	3.106–16.388	<0.001
Upper third	4.155	1.732–9.962	<0.001

OR, odd ratio; C.I, confidence interval

**Table 3:** Effects of the predictive factors associated with non-curative endoscopic resection (factors; female, lesions size ≥ 20 mm, longer procedure time ≥ 30 min, surface ulceration, surface nodularity, surface depression, undifferentiated histology and lesion located at the mid or upper third of stomach).

No. of risk factors	Non-curative resection (n = 96)	Curative resection (n = 500)	Total (n = 596)
0	1 (1.0%)	90 (18.0%)	91 (15.3%)
1	9 (9.4%)	138 (27.6%)	147 (24.7)
2	15 (15.6%)	147 (29.4)	162 (27.2)
3	19 (19.8)	85 (17.0)	104 (17.4)
4	29 (30.2)	33 (6.6)	62 (10.4)
5	19 (19.8)	7 (1.2)	26 (4.4)
6	4 (4.2)	0 (0%)	4 (0.7%)

### Clinical outcomes after non-curative endoscopic resection

Among the 96 non-curative endoscopic resections, 36 patients underwent surgical gastrectomy; lymph node metastasis was found in 28 of these patients. During follow-up examinations without gastrectomy, local recurrence was found in 2 patients in whom an additional ESD was successful. Gastric cancer related death occurred in one patient. His primary EGC was a deep, submucosal, invasive cancer with lymphovascular invasion. He refused an additional gastrectomy and a distant hepatic metastasis was found 2 years after the ESD.

## Discussion

In the present study, non-curative endoscopic resection occurred in 16.1% of patients. In order for endoscopic resection of EGC to be curative, lymph node metastasis should be absent. A previous study by Gotoda *et al*. reported that the risk of lymph node metastasis was zero in patients with EGC that met the expanded indication [11]. The reported 5-year overall survival rate of ESD for EGC, which met expanded indication, was over 95% [3,4]. However, if the pathologic result of ESD is non-curative, surgical gastrectomy should be recommended because the patient’s risk of either recurrence or lymph node metastasis might be higher than in curative resected patients. In the present study, one patient died due to gastric cancer. Although we recommend a surgical gastrectomy, he refused to undergo further surgical treatment, and died 2 years after ESD due to hepatic metastasis. In the present study, among the 35 patients who underwent surgical gastrectomy, 28 patients had lymph node metastasis even though abdominal computed tomography showed no evidence of lymph node metastasis. Therefore, if the final pathologic results of ESD are non-curative, surgical gastrectomy should be recommended even if no evidence of lymph node enlargement is found on imaging study.

In the present study, we aimed to evaluate the predictive factors associated with non-curative endoscopic resection. The possible predictive factors might be associated with submucosal invasive cancers or those difficult to remove en-bloc. In the present study, female sex, larger lesion size (≥ 20 mm), longer procedure time, ulceration, nodularity, depression, undifferentiated carcinoma, and lesions located at either the mid or upper third of stomach were all predictive factors. Patient with more risk factors had an increased risk of non-curative endoscopic resection. Therefore, if a patient has multiple predictive factors, clinicians need to be more careful when deciding on the endoscopic treatment of EGC.

To achieve curative endoscopic resection, proper selection of patients is required. Since lymph node metastasis is associated with the invasion depth of gastric cancer, prediction of submucosal cancer is important. In the past, it has been difficult to differentiate between mucosal cancer and submucosal invasive cancer. Various conventional endoscopic findings associated with submucosal invasive cancers, such as large lesion size, undifferentiated histology, irregular surface, submucosal tumor-like marginal elevation, and clubbing/abrupt cutting/fusion of converging folds have been reported [12–14]. However, the reported discrimination rate was 73.7–78.0% compared to conventional endoscopy findings [12–14]. Although endoscopic ultrasound has been used to determine the depth of invasion before resection, the reported accuracy of discrimination between mucosal and submucosal cancer was 67–85% [12–14]. In the present study, similar endoscopic appearances, such as a larger lesion size (≥ 20 mm), ulceration, nodularity, and depression, which were known factors associated with submucosal invasion, were also associated with non-curative ESD [12–14].

Non-curative endoscopic resection is associated with piecemeal resection and lateral margin involvement. Therefore, to achieve curative endoscopic resection, the ESD should be performed by a skilled endoscopist. In the present study, undifferentiated carcinoma, longer procedure time, and location of the lesion (mid or upper third of stomach) were all important predictive factors. The growth pattern of the undifferentiated carcinoma might be somewhat different from that of the differentiated carcinoma. Undifferentiated carcinoma, especially signet ring cell carcinoma, often spreads subepithelially [15]. Therefore, a larger safety lateral margin is necessary for undifferentiated carcinoma during ESD. Location of EGC may be related with various risks. The submucosa-invasive EGCs were reported more frequently in the mid/upper third stomach compared with the antrum [16]. Early detection of EGC located in the mid/upper third of stomach is more difficult than in the lower third of the stomach. Most of the EGCs located in the mid/upper third of stomach are detected using a retroflexed endoscope, and therefore, an endoscopic forceps biopsy is difficult because a front endoscopic view is difficult to maintain. The lumen of the mid/upper third of the stomach is wider than the lower third, and EGC can be hidden between the gastric folds. Therefore, EGCs located at the upper/mid third of stomach might be more easily missed or diagnosed later than those in the lower third of stomach. The thickness of the submucosa has been reported to be thinner in the body than at the antrum [17], and the lymphatic capillaries are present just above the muscularis mucosa [18]. These anatomical factors may be associated with the submucosal invasive EGC located at the upper/mid third of the stomach as an increased risk for non-curative endoscopic resection. In addition, difficult ESD might be associated with EGC location and longer procedure time. To perform ESD for EGC in the upper/mid third of stomach, handing endoscopic electrosurgical knives might be used along with endoscopic retroflexion. Patient factors, such as frequent belching during endoscopic retroflexion can make performing ESD more difficult.

The present study has several limitations. First, a retrospectively conducted study may have selection bias, and the sample size may be too small to generalize the present study results. However, the results of our study are consistent with those of other studies. Data from multicenter, prospective studies will be needed. Secondly, the mean follow-up period might be too short to determine the clinical outcomes of the patients with non-curative endoscopic resection without surgical gastrectomy.

In summary, the non-curative endoscopic resection rate was 16.1%. Surgical gastrectomy should be recommended for patients with non-curative endoscopic resection. To avoid unnecessary endoscopic treatment, we should be aware of the predictive factors for non-curative endoscopy. In the present study, female sex, larger lesion size (≥ 20 mm), longer procedure time, ulceration, nodularity, depression, undifferentiated carcinoma, and lesion located at the mid/upper third of stomach were all predictive factors. In addition, as the risk factors increased, the risk of non-curative ESD also increased. For patients with multiple risk factors, clinicians should focus on careful selection of the appropriate treatment approach for EGC before considering ESD.

## References

[1] Japanese Gastric Cancer Association. Japanese Classification of Gastric Carcinoma - 2nd English Edition. Gastric Cancer. 1998;1:10–24. 10.1007/s101209800016 PMID: 11957040 11957040

[2] Ono H. Early gastric cancer: diagnosis, pathology, treatment techniques and treatment outcomes. Eur J Gastroenterol Hepatol. 2006;18:863–836. 16825902 10.1097/00042737-200608000-00009

[3] Min BH, Kim ER, Kim KM, Park CK, Lee JH, Rhee PL, et al Surveillance strategy based on the incidence and patterns of recurrence after curative endoscopic submucosal dissection for early gastric cancer. Endoscopy. 2015;47:784–793. 10.1055/s-0034-1392249 26111362

[4] Ahn JY, Jung HY, Choi KD, Choi JY, Kim MY, Lee JH, et al Endoscopic and oncologic outcomes after endoscopic resection for early gastric cancer: 1370 cases of absolute and extended indications. Gastrointest Endosc. 2011;74:485–493. 10.1016/j.gie.2011.04.038 21741645

[5] Suh M, Choi KS, Lee YY, Jun JK. Trends in Cancer Screening Rates among Korean Men and Women: Results from the Korean National Cancer Screening Survey, 2004–2012. Cancer Res Treat. 2013;45:86–94. 10.4143/crt.2013.45.2.86 . 23864841 PMC3710967

[6] Japanese Gastric Cancer Association. Japanese gastric cancer treatment guidelines 2014 (ver. 4). Gastric Cancer. 2017;20:1–19. 10.1007/s10120-016-0622-4 PMID: 27342689 PMC5215069 27342689

[7] Kim EH, Park JC, Song IJ, Kim YJ, Joh DH, Hahn KY, et al Prediction model for non-curative resection of endoscopic submucosal dissection in patients with early gastric cancer. Gastrointest Endosc. 2017;85:976–983. 10.1016/j.gie.2016.10.018 27756614

[8] Participants in the Paris Workshop. The Paris endoscopic classification of superficial neoplastic lesions: esophagus, stomach, and colon: November 30 to December 1, 2002. Gastrointest Endosc. 2003;58:S3–43. 10.1016/S0016-5107(03)02159-X 14652541

[9] Japanese Gastric Cancer Association. Japanese classification of gastric carcinoma: 3rd English edition. Gastric Cancer. 2011;14:101–112. 10.1007/s10120-011-0041-5 PMID: 21573743 21573743

[10] Mihara M, Haruma K, Kamada T, Komoto K, Yoshihara M, Sumii K, et al The role of endoscopic findings for the diagnosis of Helicobacter pylori infection: evaluation in a country with high prevalence of atrophic gastritis. Helicobacter. 1999;4:40–48. http://doi.org.ssl.eproxy.pusan.ac.kr/10.1046/j.1523-5378.1999.09016.x 10352086 10.1046/j.1523-5378.1999.09016.x

[11] Gotoda T, Yanagisawa A, Sasako M, Ono H, Nakanishi Y, Shimoda T, et al Incidence of lymph node metastasis from early gastric cancer: estimation with a large number of cases at two large centers. Gastric Cancer. 2000;3:219–225. http://doi.org.ssl.eproxy.pusan.ac.kr/10.1007/PL00011720 11984739 10.1007/pl00011720

[12] Choi J, Kim SG, Im JP, Kim JS, Jung HC, Song IS. Endoscopic prediction of tumor invasion depth in early gastric cancer. Gastrointest Endosc. 2011;73:917–927. 10.1016/j.gie.2010.11.053 21316050

[13] Choi J, Kim SG, Im JP, Kim JS, Jung HC, Song IS. Comparison of endoscopic ultrasonography and conventional endoscopy for prediction of depth of tumor invasion in early gastric cancer. Endoscopy. 2010;42:705–713. 10.1055/s-0030-1255617 20652857

[14] Tsujii Y, Kato M, Inoue T, Yoshii S, Nagai K, Fujinaga T, et al Integrated diagnostic strategy for the invasion depth of early gastric cancer by conventional endoscopy and EUS. Gastrointest Endosc. 2015;82:452–459. 10.1016/j.gie.2015.01.022 25841580

[15] Kim H, Kim JH, Lee YC, Kim H, Youn YH, Park H, et al Growth Patterns of Signet Ring Cell Carcinoma of the Stomach for Endoscopic Resection. Gut Liver. 2015;9:720–726. 10.5009/gnl14203 25473081 PMC4625700

[16] Kang DH, Choi CW, Kim HW, Park SB, Kim SJ, Nam HS, et al Location characteristics of early gastric cancer treated with endoscopic submucosal dissection. Surg Endosc. 2017;31:4673–4679. 10.1007/s00464-017-5534-9 28389793

[17] Park S, Chun HJ, Kwon YD, Keum B, Seo YS, Kim YS, et al Stretching Causes Extensive Changes of Gastric Submucosa: Is It Acceptable to Define 500 microm as the Safe Margin? Gut Liver. 2008;2:199–204. 10.5009/gnl.2008.2.3.199 20485647 PMC2871639

[18] Akashi Y, Noguchi T, Nagai K, Kawahara K, Shimada T. Cytoarchitecture of the lamina muscularis mucosae and distribution of the lymphatic vessels in the human stomach. Med Mol Morphol. 2011;44:39–45. 10.1007/s00795-010-0503-6 21424936

